# Major Dietary Patterns among Female Adolescent Girls of Talaat Intelligent Guidance School, Tabriz, Iran

**Published:** 2012-07-30

**Authors:** M Alizadeh, J Mohtadinia, B Pourghasem-Gargari, A Esmaillzadeh

**Affiliations:** 1 Center for Food Sciences and Nutrition, Department of Nutrition and Biochemistry, School of Medicine, Urmia University of Medical Sciences, Urmia, Iran; 2 Department of Food Science and Technology, Tabriz University of Medical Sciences, Tabriz, Iran; 3 Department of Biochemistry and Dietetics, Nutrition Research Center, School of Health and Nutrition, Tabriz University of Medical Sciences, Tabriz, Iran; 4 Food Security Research Center, Department of Community Nutrition, School of Nutrition and Food Science, Isfahan University of Medical Sciences, Isfahan, Iran

**Keywords:** Dietary patterns, Factor analysis, Adolescents, Girls, Dietary intake

## Abstract

**Background:**

Increasingly nutritional experts express the necessity of research on dietary patterns to identify numerous modifiable risk factors of disease. This study was conducted to identify major dietary patterns among adolescent girls in Talaat intelligent guidance school, Tabriz, Iran.

**Methods:**

Among 257 adolescent girls aged 11-15 years, usual dietary intakes were assessed using a 162-item semi-quantitative food frequency questionnaire (FFQ). Factor analysis was used to identify major dietary patterns in this Turkish population.

**Results:**

We identified 6 major dietary patterns:(1) Western pattern high in pizza, meats and fruit juice; (2) Sweat junk foods pattern high in dried fruits, jams, honey and sugar; (3) Asian pattern high in legumes, potato and other vegetables; (4) Salty junk foods pattern high in carrot, puffs and potato chips and (6) Iranian traditional dietary pattern high in hydrogenated fats, garlic and broth.

**Conclusion:**

Our findings suggested that among the 6 major dietary patterns, Asian-like food was the healthiest one.

## Introduction

The studies in nutritional epidemiology has emphasized on the role of nutrients and foods but the entire effect of foods can only be seen by considering them as dietary patterns.[1] Increasingly, nutritional experts express the necessity of research on dietary patterns to identify numerous modifiable risk factors of food related diseases.[2][3][4]

Most studies have focused on dietary patterns among adults but data on food intake patterns of children and adolescents are scarce. This is particularly relevant for Asian countries, where we are aware of no study to report major dietary patterns among adolescents. Although some data shows similar dietary patterns between adults and adolescents in western population,[5] it remains unknown if this would be the case for adolescents in Asian countries.

Dietary patterns are major determinants of several chronic diseases[6] like obesity,[7] metabolic syndrome,[8] diabetes[9] and cardiovascular diseases.[10] Previous studies have shown that the prevalence of obesity and the metabolic syndrome among Iranian adolescents is as much as that in their counterparts in US.[11] To help prevent the increasing trend of these non-communicable diseases, the first step is to identify major dietary patterns among adolescents. This study was conducted to identify major dietary patterns among adolescent girls in Talaat Intelligent Guidance School in Tabriz, Iran.

## Materials and Methods

This cross-sectional study (From April to July 2007) was conducted in Talaat Intelligent Guidance School among 257 adolescent girls (compromising all students in the school) aged 11-15 years. Students of this school came from all districts of Tabriz. The project was approved by the Ethics Committee of the School of Nutrition and Health, Tabriz University of Medical Sciences and informed written consent was obtained from each participant. Usual dietary intakes were assessed by using a validated 162-item semi quantitative food frequency questionnaire (FFQ). Participants were asked to report the frequency of consumption of a given food item during the previous year. The reported frequency for each food item was then converted to a daily intake. Foods from FFQ were classified into 40 food groups (Table 1). Validation study of this FFQ in previous studies had shown that this questionnaire could evaluate long-term dietary intakes reasonably.[12][13]

**Table 1:** Food grouping used in factor analysis.

**Food groups**	**Food items**
Processed meats	Sausages, hamburger
Red Meat	Beef, lamb
Organ meats	Beef liver
Fish	Canned tuna fish, other fish
Poultry	Chicken with or without skin
Eggs	Eggs
Butter	Butter
Margarine	Margarine
Low fat dairy	Skim or low-fat milk, low-fat yogurt
High fat dairy	High-fat milk, whole milk, chocolate milk, cream, high-fat yogurt, cream, yogurt, cream cheese, other cheeses, ice cream
Tea	Tea
Coffee	Coffee
Fruits	Pears, apricots, cherries, apples, raisins or grapes, bananas, cantaloupe, watermelon, oranges, grapefruit, kiwi, strawberries, peaches, nectarine, tangerine, mulberry, plums, persimmons, pomegranates, lemons, pineapples, fresh figs and dates
Fruit juices	Apple juice, orange juice, grapefruit juice, other fruit juices
Cruciferous vegetables	Cabbage, cauliflower, Brussels sprouts, kale
Yellow vegetables	Carrots
Tomato	Tomatoes, tomato sauce, tomato pasta
Green leafy vegetables	Spinach, lettuce
Other vegetables	Cucumber, mixed vegetables, eggplant, celery, green peas, green beans, green pepper, turnip, corn, squash, mushrooms, onions
Legumes	Beans, peas, lima beans, broad beans, lentils, soy
Garlic	Garlic
Potato	Potatoes
Whole grain	Dark breads (Iranian), barley bread, popcorn, cornflakes, wheat germ, bulgur
Refined grains	White breads (lavash, baguettes), noodles, pasta, rice, toasted bread, milled barley, sweet bread, white flour, starch, biscuits
Pizza	Pizza
Snacks	Potato chips, corn puffs, crackers, popcorn
Nuts	Peanuts, almonds, pistachios, hazelnuts, roasted seeds, walnuts
Mayonnaise	Mayonnaise
Dried fruits	Dried figs, dried dates, dried mulberries, other dried fruit
Olive	Olives, olive oils
Sweets and desserts	Chocolates, cookies, cakes, confections
Hydrogenated fats	Hydrogenated fats, animal fats
Non-hydrogenated fats	Vegetable oils (except for olive oil)
Sugars	Sugars, candies, gaz (an Iranian confectionery made of sugar, nuts, and tamarisk)
Condiments	Jam, jelly, honey
Soft drinks	Soft drinks
Dough (Yoghurt drink)	Dough (Yoghurt drink)
Broth	Broth
Salt	Salt
Pickle	Pickle

To identify major dietary patterns, we used principal component analysis, and the factors were rotated by varimax rotation. The natural interpretation of the factors in conjunction with Eigen values>1.5 determined whether a factor should be considered as major dietary patterns. We used SPSS software (version 9.05; SPSS Inc, Chicago IL) for all statistical analyses.

## Results

We identified six major dietary patterns by the use of factor analysis (Table 2): "Western-like pattern" that was high in pizza, organ meats, fruit juice, sweats and desserts, high-fat dairy products, poultry, processed meats, fruits, refined grains, low-fat dairy products, pickles and olive. "Sweet junk foods pattern" that was highly loaded with dried fruits, jams and honey, sugars, tea, sweats and desserts, fruit juice, egg, nuts, coffee, fruit and mayonnaise. "Asian-like pattern" greatly loaded with legumes, potato, other vegetables, dough, high-fat dairy products, margarine, refined and whole grains, low-fat dairy products, egg and butter. "Salty junk foods pattern" was high in carrot, puffs, Potato chips, popcorn, crackers, pickles, coffee, tomatoes and mayonnaise. "Low protein- soft drinks-oil pattern" was high in cruciferous vegetables, green leafy vegetables, soft drinks, tomatoes, other vegetables, vegetable oils, mayonnaise and dough and finally "Iranian traditional dietary pattern" was high in hydrogenated fats, garlic, broth, tea, poultry and red meats, respectively. These factors totally explained 39.4% of the variance.

**Table 2:** Factor-loading^a^ matrix form major dietary patterns.

	**Western-like**	**Sweat junk foods**	**Asian-like**	**Salty junk foods**	**Low protein- soft drinks-oil**	**Traditional**
Pizza	0.66	-	-	0.25	-	-
Organ meats	0.60	-	-	-	-	-
Fruit juices	0.60	0.36	-	-	0.237	-
Sweets and desserts	0.58	0.37	-	-	-	-
High fat dairy	0.48	-	0.46	-	-	-
Poultry	0.46	-	-	-	-	0.42
Processed meats	0.46	0.20	-	0.26	-	-
Fruits	0.45	0.33	0.26	-	-	-
Refined grains	0.42	-	0.39	-	-	-
Low fat dairy	0.36		0.36	-	-	-
Pickle	0.34	-	-	0.38	-	-
Olive	0.33	-	-	-	0.22	-
Nuts	0.29	0.34	-	-	0.23	-
Coffee	0.28	0.33	-	0.37	0.26	-
Snacks	0.27	-	-	0.80	-	-
Butter	0.25	-	0.34	0.29	-	0.27
Sugars	0.25	0.60	-	-	-	0.24
Dough (Yoghurt drink)	0.24	-	0.48	-	0.30	-
Whole grain	0.22	-	0.37	-	-	-
Mayonnaise	0.22	0.30	-	0.33	0.31	-
Margarine	-0.21		0.41	0.27	-	-
Red meat	-	0.20	0.31	-	-0.20	0.30
Fish	-	-	-	-	-	-
Eggs	-	0.35	0.36	-	-	-
Tea	-	0.43	-	-	-	0.47
Cruciferous vegetables	-	-	-	-	0.58	-
Yellow vegetables	-	-	-	0.83	-	-
Tomato	-	-	0.20	0.34	0.42	0.29
Green leafy vegetables	-	0.26	0.25	-	0.58	-
Other vegetables	-	0.21	0.51	-	0.35	0.249
Legumes	-	-	0.62	-	-	-
Garlic	-	-	-	-	0.21	0.51
Potato	-	-	0.57	-	-	-
Dried fruits	-	0.74	-	-	-	-
Hydrogenated fats	-	-	-	-	-	0.58
Non-hydrogenated fats	-	-	-	-	0.34	-
Condiments	-	0.63	-	-	-	-
Soft drinks	-	-	-	0.26	0.49	-
Broth	-	-	-	-	-	0.47
Salt	-	-	-	-	-	-
Percent of variance explained	16.6	5.6	4.7	4.4	4.0	3.8

^a^ Factor loadings <0.2 are omitted for simplicity. Total variance explained by six factors: 39.4.

## Discussion

Few data are available about dietary patterns of adolescents. Shin et al.[14] reported 3 major dietary patterns: (1) Korean healthy pattern; (2) Animal foods pattern and (3) Sweats pattern. Korean healthy, animal foods and sweats patterns are similar to Asian-like, western-like and sweat junk foods patterns in our study, respectively. McNaughton et al.[15] identified 3 dietary patterns labeled a fruit, salad, cereals and fish pattern; a high fat and sugar pattern; and a vegetable pattern. Marques et al.[16] identified four distinct dietary patterns: The first pattern was characterized by an energy-dense diet, the second pattern represented a healthy diet, the third pattern represented intake of soft drinks and the fourth pattern represented a diet rich in calories and sugars. Lozada et al.[17] identified 4 major dietary patterns: Pattern 1 had a positive loading factor on wheat products, desserts, and meat; pattern 2 was characterized by a high consumption of low-fat dairy and low-fiber breakfast cereals; pattern 3 had a high loading for sweetened beverages and industrialized foods and pattern 4 had a moderate loading on maize products and legumes. Ritchie et al.[5] showed that healthy pattern was characteristic of high intake of fruits, vegetables, dairy, grains without added fats, mixed dishes and soups, and a low intake of sweetened drinks, other sweets, fried foods, burgers, and pizza. These patterns are different from those obtained in our study. This can be explained by demographic, cultural and racial differences.

Dietary patterns among children and adolescents are not the same across different studies while mostly the same patterns have been reported for adults.[1][8][18][19][20] This might be explained by adult attempts in adhering to a healthy lifestyle, while dietary patterns of adolescents can represent their selection of taste, family economical and cultural status. Western-like dietary pattern explains the most variance (16.6%), while traditional Iranian dietary pattern explains the least variance (3.8%). This finding indicates society transition from traditional to processed and western foods. As mentioned, traditional Iranian dietary pattern has less processed foods, while most of the foods in Western-like dietary pattern are processed foods. Although we can not call traditional Iranian dietary pattern as ideal pattern, in the same way, transition to Western-like pattern is not acceptable, too. Sweat junk foods pattern is the second pattern which explained high variance of the total diet. This pattern contains junk foods instead of main foods. High intakes of dried fruits, egg, nuts and fruits in sweat junk foods pattern represent this message that we can manage nutrition in adolescents with high intake of junk foods and gradually reduce simple sugars and replace them with dried fruits, nuts and fruit. Third pattern is Asian-like pattern which is the healthiest pattern among six patterns we identified. Subjects adhering this pattern were lactoovo-vegetarian with low intakes of meats and meat products. Asian-like pattern was high in carbohydrate and low in animal foods. Adding fruits combined with reducing refined cereals will make this dietary pattern as an ideal healthy dietary pattern. Salty junk foods pattern is the next dietary pattern. Most of foods loaded in this dietary pattern have similar tastes of salty; however adolescents adhering this pattern were eating carrot as much as puffs, chips, popcorn and cracker. Low protein-soft drinks-oil dietary pattern is fifth dietary pattern which is lowest in protein and highest in non-hydrogenated fats. Consumption of this dietary pattern was simultaneously associated with higher intakes of beverages such as soft drinks and dough, salad and mayonnaise. High consumption of vegetables and non-hydrogenated fats represents high consumption of fried vegetables such as squash, eggplant, celery, onion and mushroom. Simultaneous high consumption of vegetables, soft drinks and oil is complex paradox, which may be due to wrong nutritional knowledge or special taste. The last pattern is traditional Iranian dietary pattern which is high in hydrogenated fats, garlic, broth, tea, poultry and meats.

Several limitations need to be considered in the interpretation of our findings. First, we assessed dietary patterns by using food intake data only, whereas the inclusion of eating behaviors such as meal and snack patterns in dietary pattern analysis has been recommended.[21] Second, like any other measurements, dietary assessment also has its own measurement errors. Third, limitations of factor analysis that originate from several subjective or arbitrary decisions should also be taken into account.[22] Fourth, we can not generalize our findings to all adolescents in the country, because dietary intakes and other lifestyle measures in Tabriz who are a Turkish population are somewhat different from those in other parts of the country. Moreover, these dietary patterns are confined to adolescent girls. Furthermore, the uniform background of the study participants in terms of intelligence, sex, and education limits the extent to which we may generalize our findings. Our findings suggest that among the six major dietary patterns in Tabrizi adolescent girls: Asian-like pattern was the healthiest pattern among this population.
